# 33. STIGMA AND RECOVERY AMONG YOUNG PEOPLE AT RISK FOR PSYCHOSIS: NOVEL INSIGHTS FROM GLOBAL RESEARCH

**DOI:** 10.1093/schbul/sby014.136

**Published:** 2018-04-01

**Authors:** Sara Evans-Lacko

**Affiliations:** London School of Economics and Political Science

## Abstract

**Overall Abstract:** The recognition that attenuated clinical symptoms and impaired functioning often precede the onset of psychotic disorders has led to potentially transformative early intervention strategies which could benefit individuals during the prodromal phase through clinical intervention. However, these efforts could present a double-edged sword whereby on one hand earlier treatment could improve symptoms and facilitate recovery, but on the other hand, it could also increase labeling and associated stigma. This set of presentations draws from a diverse set of countries and researcher backgrounds, including peer perspectives to enhance recovery. Our panelists present novel data to address the potential challenges of early intervention strategies and how a better understanding of the stigmatising beliefs and experiences among individuals during the earliest signs of psychotic disorder could improve early intervention efforts.

Two presentations use new perspectives to evaluate the perceptions and impact of stigma among prospective cohorts of young people at elevated risk for psychosis. Lawrence Yang uses one of the largest known cohorts of clinical high-risk young people from a multi-site study in the USA to assess the impact of two types of stigma: stigma associated with symptoms and and stigma associated with labelling of the ‘high-risk for psychosis’ identification. Sara Evans-Lacko uses two unique prospective community cohorts of young people enriched for risk of psychotic disorder from Brazil and the UK and investigates the levels of personal stigma and mental health literacy in relation to psychosis among young people with and without high risk of developing psychotic disorder. She will then present new data on how this relates to intended help-seeking and actual mental health service use among those at-risk for psychotic disorder. Utilizing a different perspective based on changing medical terminology for at-risk states, Danny Koren explores the attributions made by individuals in the general population and mental health professionals when applying ‘attenuated pathology’ versus ‘compromised health’ labels to refer to at-risk psychosis states.

